# Modulation of the Substitution Pattern of 5-Aryl-2-Aminoimidazoles Allows Fine-Tuning of Their Antibiofilm Activity Spectrum and Toxicity

**DOI:** 10.1128/AAC.00035-16

**Published:** 2016-10-21

**Authors:** Elien Peeters, Geert Hooyberghs, Stijn Robijns, Kai Waldrant, Ami De Weerdt, Nicolas Delattin, Veerle Liebens, Soňa Kucharíková, Hélène Tournu, Natalie Verstraeten, Barbara Dovgan, Lenart Girandon, Mirjam Fröhlich, Katrijn De Brucker, Patrick Van Dijck, Jan Michiels, Bruno P. A. Cammue, Karin Thevissen, Jozef Vanderleyden, Erik Van der Eycken, Hans P. Steenackers

**Affiliations:** a Centre of Microbial and Plant Genetics (CMPG), Department of Microbial and Molecular Systems, KU Leuven, Leuven, Belgium; b Laboratory for Organic & Microwave-Assisted Chemistry (LOMAC), Department of Chemistry, KU Leuven, Leuven, Belgium; c Department of Molecular Microbiology, VIB, Leuven, Belgium; d Laboratory of Molecular Cell Biology, Department of Biology, KU Leuven, Leuven, Belgium; e Educell, Trzin, Slovenia; f Faculty of Medicine, Institute of Cell Biology, University of Ljubljana, Ljubljana, Slovenia

## Abstract

We previously synthesized several series of compounds, based on the 5-aryl-2-aminoimidazole scaffold, that showed activity preventing the formation of Salmonella enterica serovar Typhimurium and Pseudomonas aeruginosa biofilms. Here, we further studied the activity spectrum of a number of the most active *N*1- and 2*N*-substituted 5-aryl-2-aminoimidazoles against a broad panel of biofilms formed by monospecies and mixed species of bacteria and fungi. An *N*1-substituted compound showed very strong activity against the biofilms formed by Gram-negative and Gram-positive bacteria and the fungus Candida albicans but was previously shown to be toxic against various eukaryotic cell lines. In contrast, 2*N*-substituted compounds were nontoxic and active against biofilms formed by Gram-negative bacteria and C. albicans but had reduced activity against biofilms formed by Gram-positive bacteria. In an attempt to develop nontoxic compounds with potent activity against biofilms formed by Gram-positive bacteria for application in antibiofilm coatings for medical implants, we synthesized novel compounds with substituents at both the *N*1 and 2*N* positions and tested these compounds for antibiofilm activity and toxicity. Interestingly, most of these *N*1-,2*N*-disubstituted 5-aryl-2-aminoimidazoles showed very strong activity against biofilms formed by Gram-positive bacteria and C. albicans in various setups with biofilms formed by monospecies and mixed species but lost activity against biofilms formed by Gram-negative bacteria. In light of application of these compounds as anti-infective coatings on orthopedic implants, toxicity against two bone cell lines and the functionality of these cells were tested. The *N*1-,2*N*-disubstituted 5-aryl-2-aminoimidazoles in general did not affect the viability of bone cells and even induced calcium deposition. This indicates that modulating the substitution pattern on positions *N*1 and 2*N* of the 5-aryl-2-aminoimidazole scaffold allows fine-tuning of both the antibiofilm activity spectrum and toxicity.

## INTRODUCTION

Biofilms are complex, condition-dependent, surface-associated communities of microorganisms embedded in a self-produced matrix (1–4). The bacteria within biofilms are up to 1,000 times more tolerant of antibiotics, disinfectants, and other stress factors, and this tolerance strongly impedes antimicrobial treatment (5). Hence, persistent biofilm infections and contaminations often occur and cause a tremendous amount of problems in various sectors, including the medical, food industry, household, and agricultural sectors (6–8). In the medical sector, biofilms are often associated with implantable devices (9–12). Staphylococci are the principal microorganisms that colonize these devices. They comprise up to two-third of all pathogens in orthopedic implant infections, where they can cause septic arthritis and osteomyelitis, resulting in the inflammatory destruction of bones and joints (13). The dimorphic fungus Candida albicans, the most frequent cause of candidiasis, is also often associated with the formation of biofilms on the surface of medical devices and tissues in general (14).

Given the extent of problems caused by biofilms, there has been a strong effort to develop novel antibiofilm strategies (15–19). One of the most promising approaches is the use of compounds able to prevent or eradicate biofilms without affecting the planktonic growth of the microorganisms (20, 21). These specific antibiofilm compounds are believed to be less prone to resistance development. They could be used in several applications, one of which is as antibiofilm coatings on the surface of implantable medical devices, such as orthopedic implants or dental implants (22–25).

**FIG 1:**
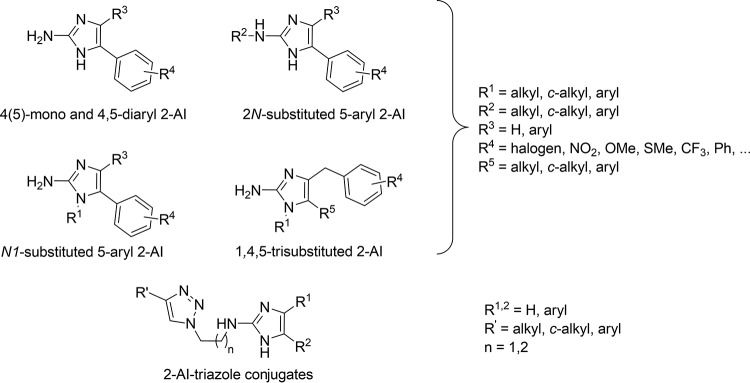
Classes of 5-aryl-substituted 2-AIs with antibiofilm activity reported by our group (26–29). OMe, methoxy group, SMe, methylthio group; Ph, phenyl group.

We have previously reported on several series of specific antibiofilm compounds based on the 2-aminoimidazole (2-AI) scaffold. As illustrated in Fig. 1, these series include the monosubstituted 5-aryl-2-AIs (5-Ar-2-AIs) (26), *N*1-substituted 5-Ar-2-AIs (26), 2*N*-substituted 5-Ar-2-AIs (27), 4,5-disubstituted 2-AIs (26), 1,4,5-trisubstituted 2-AIs (28), and 2-AI–triazole conjugates (29). These compounds were shown to display activity preventing the formation of biofilms of Salmonella enterica serovar Typhimurium, one of the most important causes of foodborne infections worldwide and a notorious biofilm former both inside and outside the host, and of Pseudomonas aeruginosa, an opportunistic Gram-negative bacterial pathogen that can infect immunocompromised people, such as cystic fibrosis patients, and cause life-threatening chronic lung infections (30). Moreover, P. aeruginosa biofilms can occur on a variety of medical devices, such as intravascular and urinary catheters. The molecular mechanism of the antibiofilm activity of 5-phenyl-2-aminoimidazole was studied in *S*. Typhimurium (31). It was shown that this compound reduces the transcription of CsgD, the master regulator of biofilm formation, and its regulon genes, *csgB* and *adrA* (involved in curli and cellulose production, respectively [32]), during the first 24 h of biofilm formation. This indicates that under the influence of the compound, Salmonella forms fewer biofilm matrix components, thereby at least partly explaining the inhibitory mode of action of the 2-aminoimidazoles.

**FIG 2:**
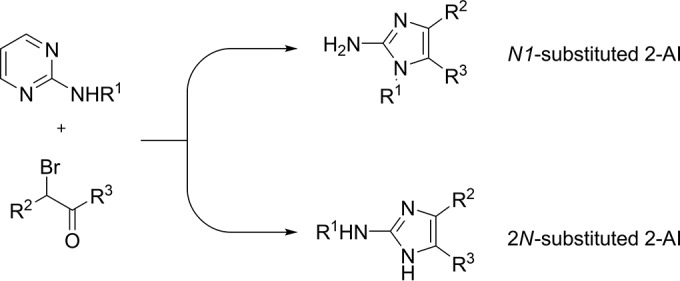
Diversely oriented approach toward 2-AIs developed by our research group (34, 94). R^1^ = alkyl, c-alkyl, aryl; R^2^ = H, aryl; R^3^ = aryl.

During the past decade, several synthetic methodologies leading to diversely substituted 2-AIs have been published (28, 29, 33–35). Our research group has developed a diversely oriented approach toward 2-AIs from 2-aminopyrimidines and α-bromoketones, as shown in Fig. 2. By switching reaction conditions, the selective synthesis of either *N*1-substituted 2-AIs or 2*N*-substituted 2-AIs can be achieved.

In the search for new antibiofilm compounds, most attention has been focused on monospecies biofilms. However, it has become clear that in nature biofilms often consist of more than one microbial species (36–41). For instance, it is estimated that 27% of nosocomial C. albicans bloodstream infections are polymicrobial, with Staphylococcus aureus being the third most common organism isolated in conjunction with C. albicans (42, 43). Within mixed biofilms, bacteria preferably interact with hyphal C. albicans cells (44, 45). Mixed-species biofilms are often more resilient than single-species biofilms, which has further implications for their control and manipulation in a variety of applications (36, 37, 46–53). In mixed biofilms of S. aureus and C. albicans cells, for instance, the S. aureus cells show enhanced resistance to vancomycin, an effect which is in part mediated by the C. albicans matrix (47, 54). Therefore, nowadays multispecies biofilms are included in many more preclinical research activities.

In the current study, we further explored the activity spectrum of a number of the most active previously reported 2-AIs against a broad panel of monospecies and mixed-species biofilms consisting of bacteria and fungi. Our microbial test panel included S. aureus and Staphylococcus epidermidis (Gram-positive cocci), which can colonize different types of implantable devices (9), chronic wounds (4), and catheters (55, 56); Porphyromonas gingivalis (a Gram-negative bacteroidetes), an important constituent in dental plaque biofilms involved in periodontal diseases (57); Escherichia coli (a Gram-negative gammaproteobacterium) known to form biofilms on inter alia urinary catheters (4), plant material (58), and food (contact) surfaces (59); Serratia liquefaciens (a Gram-negative gammaproteobacterium), capable of colonizing a wide variety of surfaces in water, soil, the digestive tracts of rodents, plants, insects, fish, and humans (nosocomial infections) (60); Burkholderia cepacia (a Gram-negative betaproteobacterium), involved in biofilm infections in the lungs of cystic fibrosis patients (61); and C. albicans, an opportunistic fungal pathogen capable of invading any site of the human host, from deep tissues and organs to superficial sites, implants, and catheters (62), along with the previously tested bacteria *S*. Typhimurium and P. aeruginosa (Gram-negative gammaproteobacteria).

We show that the *N*1-substituted compounds have broad activity but are toxic, whereas the 2*N*-substituted compounds are nontoxic but lack a broad spectrum of activity against Gram-positive bacteria. We hypothesized that 5-Ar-2-AIs substituted at both the *N*1 and 2*N* positions might combine the broad-spectrum activity of the *N*1-substituted compounds (or at least the activity against Gram-positive bacteria) with the low toxicity of the 2*N*-substituted compounds. A series of eight *N*1-,2*N*-disubstituted 5-Ar-2-AIs was synthesized and tested for antibiofilm activity and toxicity against bone cells. A first motivation for evaluation of toxicity against bone cells is that the expected antibiofilm activity profile of these compounds makes them well suited for application in antibiofilm coatings for implants, such as orthopedic implants (11, 13). The second motivation is that it allows an easy comparison with the toxicity of the previously described 5-Ar-2-AIs, which has been evaluated using the same assays used in the present study (36). The novel compounds were indeed shown to be nontoxic and have a broad spectrum of activity against Gram-positive bacteria; however, this broad spectrum of activity was at the cost of the loss of their antibiofilm activity against Gram-negative bacteria.

## MATERIALS AND METHODS

### Chemistry.

All solvents and reagents were purchased from commercial sources and were used without prior purification. This-layer chromatography analysis was performed on aluminum-backed plates. The products were purified by silica gel (200- to 300-mesh) column chromatography. All nuclear magnetic resonance (NMR) spectra were recorded on a Bruker Avance 300 spectrometer at 300 MHz (^1^H) and at 75 MHz (^13^C). The ^1^H and ^13^C chemical shifts are reported in parts per million relative to the signal for tetramethylsilane using the residual solvent signal as the internal reference. The following abbreviations were used to designate chemical shift multiplicities: s, singlet; d, doublet; dd, doublet of doublets; t, triplet; dt, doublet of triplets; q, quartet; p, pentet; and m, multiplet. The ^13^C NMR spectra are proton decoupled. 2-Aminoimidazole compounds 1 to 7 were synthesized according to established literature procedures (27–29, 34).

### General procedure for the synthesis of *N*-substituted 2-aminoimidazole compound 8.

To a solution of 2-aminoimidazole in toluene was added isobutyraldehyde or cylcopentanon (1.2 equivalents). The mixture was stirred at 120°C for 3 h. After cooling to room temperature, the solvent was reduced *in vacuo*. The crude intermediate was dissolved in methanol and cooled to 0°C. NaBH_4_ (4 equivalents) was added portion wise. The reaction was stirred for 16 h at room temperature. The solvent was reduced *in vacuo*, and the crude product was taken up in water and extracted with ethyl acetate. The resulting organic phases were washed with brine, dried over sodium sulfate, filtered, and concentrated *in vacuo*. The products were purified by chromatography over silica gel with ethyl acetate-heptane (7:3) as the eluent.

NMR spectra are provided in the supplemental material.

### Strains and growth media.

The strains P. aeruginosa PA14 (63), Escherichia coli TG1 (64), E. coli MG1655 (65), S. enterica serovar Typhimurium ATCC 14028 (66), Porphyromonas gingivalis ATCC 33277 (67), Serratia liquefaciens MG44 (68), Burkholderia cepacia LMG1222T (69), C. albicans SC5314 (70), Staphylococcus aureus ATCC 6538, S. aureus SH1000 (71, 72), and Staphylococcus epidermidis (73) were used in this study. Overnight cultures of C. albicans SC5314 were grown with aeration in 1% yeast extract, 2% peptone, and 2% dextrose (YPD) at 30°C. Overnight cultures of E. coli TG1, *S*. Typhimurium ATCC 14028, S. liquefaciens MG44, B. cepacia LMG1222T, S. aureus ATCC 6538, S. aureus SH1000, and S. epidermidis were grown with aeration in lysogeny broth (LB) at 37°C (64). Overnight cultures of P. gingivalis ATCC 33277 were grown anaerobically (Anoxomat, AN20°; Mart Microbiology, Drachten, the Netherlands) in LB at 37°C. Overnight cultures of P. aeruginosa PA14 were grown with aeration in LB or in tryptic soy broth (TSB) at 37°C. Overnight cultures of E. coli MG1655 were grown with aeration in TSB at 37°C. Phosphate-buffered saline (PBS) was prepared by combining 8.8 g liter^−1^ NaCl, 1.24 g liter^−1^ K_2_HPO_4_, and 0.39 g liter^−1^ KH_2_PO_4_ (pH 7.4). RPMI 1640 medium with l-glutamine and without sodium bicarbonate was purchased from Sigma and buffered to pH 7.0 with MOPS (morpholinepropanesulfonic acid; Sigma, St. Louis, MO) (final concentration, 165 mM).

### Monospecies antibiofilm assays. (i) Inhibition of bacterial biofilms.

A static peg assay, described previously (27, 74), was used for bacterial biofilm formation. The Calgary biofilm device consists of a platform carrying 96 polystyrene pegs (Nunc no. 445497) that fits as a microtiter plate lid, with 1 peg hanging into each microtiter plate well (Nunc no. 269789). Twofold serial dilutions of the compounds (dissolved in 100% dimethyl sulfoxide [DMSO] or ethanol) in 100 μl liquid broth (TSB diluted 1/20) per well were prepared in the microtiter plate in duplicate or triplicate with a maximum concentration of 1,600 μM and a minimum concentration of 0.8 μM. Subsequently, an overnight culture of *S*. Typhimurium ATCC 14028, P. aeruginosa PA14, E. coli TG1, S. epidermidis, S. aureus SH1000, or S. aureus ATCC 6538 (all grown in LB) was diluted 1:100 into TSB diluted 1/20 (or TSB for S. epidermidis, S. aureus SH1000, and S. aureus ATCC 6538), whereas overnight cultures of S. liquefaciens MG44 and B. cepacia LMG1222T were diluted 1:50 into TSB diluted 1/20. P. gingivalis ATCC 33277 cultures were diluted in TSB diluted 1/20 to have a final concentration of 1 × 10^8^ cells/ml. Next, 100 μl was added to each well of the microtiter plate, resulting in a total volume of 200 μl medium per well (final concentration range of compounds, 800 μM [2% DMSO or ethanol] to 0.4 μM [0.001% DMSO or ethanol]). In the next step, the pegged lid was placed on the microtiter plate and the plate was incubated for 24 h or 48 h at 25°C or 37°C without shaking. At 37°C, the plates were placed in a sealed container with wet towels on the bottom to prevent evaporation of the growth medium. Biofilms of P. gingivalis ATCC 33277 were grown anaerobically at 37°C for 72 h. During this incubation period, biofilms were formed on the surface of the pegs. After incubation, the optical density at 600 nm (OD_600_) for the planktonic cells in the microtiter plate was measured using a Synergy MX multimode reader (BioTek, Winooski, VT). This gives a first indication of the effect of the compounds on planktonic growth. For quantification of biofilm formation, the pegs were washed once in 200 μl PBS. The remaining attached bacteria were stained for 30 min with 200 μl 0.1% (wt/vol) crystal violet in an isopropanol-methanol-PBS solution (1:1:18, vol/vol). Excess stain was rinsed off by placing the pegs in a 96-well plate filled with 200 μl distilled water per well. After air drying of the pegs (30 min), the dye bound to the adherent biofilm was extracted with 30% glacial acetic acid (200 μl per well of a 96-well plate). The optical density at 570 nm (OD_570_) of each well was measured using a Synergy MX multimode reader (BioTek, Winooski, VT). The concentration of each compound needed to inhibit biofilm formation by 50% (BIC_50_) and the concentration of each compound needed to inhibit planktonic growth by 50% (IC_50_) were determined from the concentration gradient by using nonlinear curve fitting (GraphPad Prism software, version 5; GraphPad Software, Inc., La Jolla, CA). In the same assay, the effect on planktonic growth was evaluated. The activity was considered biofilm specific if the BIC_50_ was at least two times lower than the IC_50_. Data represent the means from at least 3 technical repeats with the corresponding 95% confidence intervals (provided in the supplemental material).

### (ii) Inhibition of C. albicans biofilms.

The potential of the compounds to prevent C. albicans SC5314 biofilm formation was assessed using the CellTiter-Blue (CTB) quantification method (75). For the CTB method, an overnight culture of C. albicans SC5314 was washed with PBS and a suspension of 10^6^ cells/ml (OD_600_ = 0.1) was prepared in RPMI 1640 medium (pH 7.0). Twofold serial dilutions of the compounds (dissolved in 100% DMSO or ethanol) in 100 μl RPMI 1640 medium per well were prepared in a round-bottom polystyrene 96-well microtiter plate (TPP; Trasadingen, Switzerland) in duplicate or triplicate with a maximum concentration of 1,600 μM and a minimum concentration of 0.8 μM. One hundred microliters of the cell suspension was added to each well of the microtiter plate, resulting in a total volume of 200 μl medium per well (final concentration range of compounds, 800 μM [2% DMSO or ethanol] to 0.4 μM [0.001% DMSO or ethanol]). After 16 h of static incubation at 37°C, the biofilms were washed and quantified by the CTB method as described previously (73).

### Mixed-species antibiofilm assays. (i) E. coli-P. aeruginosa biofilms.

Overnight cultures of E. coli TG1 and P. aeruginosa PA14 were diluted 1/100 in the same vial of TSB diluted 1/20 to form a mixed-culture suspension. Next, 2-fold serial dilutions of the compounds (dissolved in 100% DMSO or ethanol) in 100 μl liquid broth (TSB diluted 1/20) per well were prepared in the microtiter plate of the Calgary biofilm device (Nunc no. 269789) in duplicate or triplicate with a maximum concentration of 1,600 μM and a minimum concentration of 0.8 μM. One hundred microliters of the mixed-culture suspension was added to each well of the microtiter plate, resulting in a total volume of 200 μl medium per well (final concentration range of compounds, 800 μM [2% DMSO or ethanol] to 0.4 μM [0.001% DMSO or ethanol]). The pegged lid was placed on the microtiter plate, and the plate was incubated for 72 h at 37°C, which allowed biofilm formation on the pegs (Nunc no. 269789) of the Calgary biofilm device. After 72 h, the biofilm was colored with crystal violet as described above (74). The OD_570_ (biofilm cells) and OD_600_ (planktonic cells) were measured, and the BIC_50_ and the IC_50_, respectively, were calculated.

### (ii) S. aureus-S. epidermidis biofilms.

Overnight cultures of S. aureus ATCC 6538 and S. epidermidis were grown in LB medium and were diluted 1/200 in the same vial of TSB to form a mixed-culture suspension. Next, 2-fold serial dilutions of the compounds (dissolved in 100% DMSO or ethanol) in 100 μl TSB medium per well were prepared in the microtiter plate (Nunc no. 269789) in duplicate or triplicate with a maximum concentration of 1,600 μM and a minimum concentration of 0.8 μM. One hundred microliters of the mixed-culture suspension was added to each well of the microtiter plate, resulting in a total volume of 200 μl medium per well (final concentration range of compounds, 800 μM [2% DMSO or ethanol] to 0.4 μM [0.001% DMSO or ethanol]). The cells were then incubated for 48 h at 37°C, which allowed biofilm formation on the pegs (Nunc no. 269789) of the Calgary biofilm device. After 24 h, fresh medium with compounds was added to the wells, and after 48 h, the biofilm was colored with crystal violet as described above (74). The OD_570_ (biofilm) and OD_600_ (planktonic) were measured, and the BIC_50_ and the IC_50_, respectively, were calculated.

### (iii) C. albicans-E. coli biofilms.

Overnight cultures of C. albicans SC5314 (YPD) and E. coli MG1655 (TSB) were washed three times with PBS, after which they were diluted in RPMI 1640 medium to OD_600_s of 1 and 0.01, respectively. Equal volumes of these cell suspensions were mixed, and 100 μl of this mixed cell suspension together with compound was added to the wells of a microtiter plate in triplicate. Concentrations of 25 μM (0.0625% DMSO or ethanol) and 100 μM (0.25% DMSO or ethanol) were tested. After 24 h of incubation at 37°C, the medium was removed and the biofilm was washed with PBS. Next, the cells were resuspended in 100 μl of PBS by scraping them off, sonicated (1 min, 45 kHz; USC300-T; VWR, Radnor, PA, USA), and vigorously pipetted up and down. Finally, dilution series were made, and quantification of the E. coli MG1655 and C. albicans SC5314 populations was performed using selective plating on tryptic soy agar (TSA) plates containing 25 mg/liter amphotericin B and YPD plates containing 100 μg/ml tetracycline, respectively. The percentage of C. albicans SC5314 and E. coli MG1655 cells relative to the number of cells after DMSO or ethanol control treatment was determined.

### (iv) C. albicans-S. epidermidis biofilms.

Overnight cultures of C. albicans SC5314 (YPD) and S. epidermidis (TSB) were diluted in RPMI 1640 medium to OD_600_s of 0.05 and 0.01, respectively. Equal volumes of the cell suspensions of each organism were mixed before use. One hundred microliters of this mixed cell suspension together with compound was added to the wells of a round-bottom microtiter plate (TPP; Trasadingen, Switzerland) in triplicate. Concentrations of 25 μM (0.0625% DMSO or ethanol) and 100 μM (0.25% DMSO or ethanol) were tested. After 24 h of incubation at 37°C, the biofilms were washed with PBS and fresh medium with or without compounds was added. After a further incubation for 48 h at 37°C, the biofilms were washed with PBS, after which the cells were resuspended in 100 μl of PBS by scraping them off, sonicated (1 min, 45 kHz; USC300-T; VWR, Radnor, PA, USA), and vigorously pipetted up and down. Finally, the biofilm cells were diluted in PBS and plated on YPD agar plates containing 100 mg/liter ampicillin and TSA plates containing 25 mg/liter amphotericin B to determine the number of fungal and bacterial CFU, respectively, after 2 days of incubation at 37°C. The percentage of C. albicans SC5314 and S. epidermidis cells relative to the number of cells after DMSO or ethanol control treatment was determined.

### (v) C. albicans-S. aureus biofilms.

Overnight cultures of C. albicans SC5314 (YPD, 30°C) and S. aureus SH1000 (LB, 37°C) were washed with PBS, after which they were diluted in RPMI 1640 medium to obtain cell suspensions of 10^6^ cells/ml for fungal cells and 10^8^ cells/ml for bacteria. Equal volumes of these cell suspensions were mixed, and 100 μl of this mixed cell suspension together with compound was added to the wells of a microtiter plate in triplicate. Concentrations of 25 μM (0.0625% DMSO or ethanol) and 100 μM (0.25% DMSO or ethanol) were tested. The plates were incubated at 37°C for 90 min. After incubation, the wells were washed twice with PBS, and 200 μl of fresh RPMI 1640 medium with or without compounds was added in triplicate to the wells. After 24 h of incubation at 37°C, the medium was removed and the biofilm was washed with PBS. Next, the cells were resuspended in 100 μl of PBS by scraping them off, sonicated (1 min, 45 kHz; USC300-T; VWR, Radnor, PA, USA), and vigorously pipetted up and down. Finally, dilution series were made, and quantification of the S. aureus SH1000 and C. albicans SC5314 populations was performed using selective plating on TSA plates containing 25 mg/liter amphotericin B and YPD plates containing 100 μg/ml tetracycline, respectively. The percentage of C. albicans SC5314 and S. aureus SH1000 cells relative to the number of cells after DMSO or ethanol control treatment was determined.

### Mammalian cell viability assay.

The viability of two human primary cell types, namely, osteoblasts (OB) and bone marrow-derived mesenchymal stem cells (MSC), was tested according to the ISO 10993-5 standard, as previously described (76). Briefly, cells were seeded in 96-well tissue culture test plates (TPP; Trasadingen, Switzerland) at 5 × 10^3^ cells/cm^2^ in cell culture medium (advanced Dulbecco modified Eagle's medium [DMEM]) supplemented with 10% serum, 1× GlutaMAX, and 0.05 mg/ml gentamicin and were allowed to attach overnight. On the next day, the cells were exposed to (i) cell culture medium and medium with the corresponding control (0.5% ethanol or DMSO; negative controls), (ii) medium with 0.05% phenol (cytotoxic control), and (iii) medium with compounds (12.5 μM) and incubated for 2 h, 48 h, and 6 days (8 repeats for each condition). At each time point, the numbers of viable and dead cells were determined directly by trypan blue staining and indirectly by measuring metabolic activity with 3-(4,5-dimethylthiazol-2-yl)-2,5-diphenyltetrazolium bromide (MTT) staining.

### (i) Trypan blue staining.

The medium was removed from the wells, 1/3 trypan blue in DMEM was added to the cells, and the cells were incubated for 3 min, after which the trypan blue was removed and DMEM was added to the wells. In each of four wells, two microscopy fields were counted for viable (transparent) and dead (blue) cells.

### (ii) MTT staining.

The medium was removed from the wells, and 100 μl of medium supplemented with 10% serum and 0.5 mg/ml MTT was added to the cells. The cells were incubated overnight at 37°C in 5% CO_2_. On the next day, the medium with MTT was removed and 100 μl acidic isopropanol was added. The cells were then centrifuged at 2,300 × *g*, and 50 μl of the supernatant was transferred to a new 96-well plate. The absorbance at 570 nm was measured, and the background at 660 nm was measured. Four wells per condition were examined.

### Osteogenic differentiation.

The effects of the substances on the osteogenic differentiation potential were assessed as previously described (76). Only the substances that allowed survival of the cells for more than 3 weeks, which is the time needed for mature osteogenic differentiation, were tested. Briefly, osteoblasts and bone marrow-derived mesenchymal stem cells were cultured in a positive solvent control (osteogenic medium with 0.5% DMSO or ethanol background), a negative control (medium without osteogenic supplements), and treated samples (osteogenic medium, 0.5% DMSO or ethanol background, and 12.5 μM test compound) with four repeats per condition. The cells in the mesenchymal stem cell and osteoblast cultures were harvested after 3 or 5 weeks, respectively, for the calcium and DNA assay.

### Calcium and DNA assay.

The calcium deposition of osteoblasts and mesenchymal stem cells was measured with the calcium CPC LiquiColor test (Stanbio Laboratory, Boerne, TX) as previously described (76). Briefly, cell cultures were extracted with 5% trichloroacetic acid (500 μl per sample), *o*-cresolphthalein complex was added, and the calcium content was determined spectrophotometrically at 550 nm. The DNA content was determined as previously described (76). DNA values were used to normalize the calcium content. Four wells per condition were examined, and two samples from each well were taken for each assay.

## RESULTS AND DISCUSSION

### Benchmarking of antibiofilm potency based on BIC_50_ values.

In order to classify the antibiofilm potency of the 5-aryl-2-aminoimidazoles (5-Ar-2-AIs) against bacterial and fungal biofilms, we compared their antibiofilm activity to the activities of three reference compounds, baicalein, nifuroxazide, and tannic acid (Table 1), to those of various antibiofilm compounds identified via in-house screenings of compound libraries (77), and to those of antibiofilm compounds previously reported in the literature (74, 78–81).

**TABLE 1:**
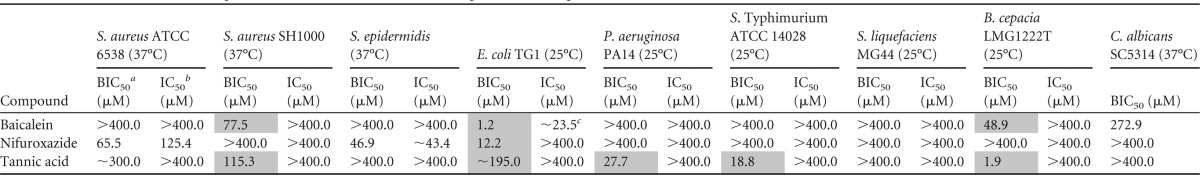
Effects of benchmark compounds described in the literature on a panel of monospecies biofilms^*d*^

a BIC_50_, concentration of compound needed to inhibit biofilm formation by 50%.

b IC_50_, concentration of compound needed to inhibit planktonic growth by 50%.

c ∼, the BIC_50_ or IC_50_ values could not be accurately calculated due to the steepness of the curve.

d Results for compounds that have biofilm-specific activity (2× BIC_50_ < IC_50_) are shaded in gray. The 95% confidence intervals are provided in Table S1 in the supplemental material.

The three reference compounds were chosen on the basis of their previously reported preventive, biofilm-specific activity, toxicity, and commercial availability: (i) baicalein at 20 μM inhibits biofilm formation of P. aeruginosa PAO1 (82), whereas biofilm formation of C. albicans SC5314 is inhibited by 10 to 100 μM baicalein (83); (ii) nifuroxazide inhibits P. aeruginosa PAO1 biofilm formation at 70 μM (84); and (iii) tannic acid inhibits the biofilm formation of S. aureus SH1000, E. coli VR50, and E. coli F18 at 20 μM (85, 86).

In this study, we found these reference compounds to be inactive or characterized by BIC_50_ values higher than 50 μM (Table 1) against their target species mentioned above, emphasizing the stringency of the thresholds used and the importance of the test conditions and the specific strains used. Remarkably, however, all three reference compounds displayed antibiofilm activities against a number of other species. We found that baicalein displayed antibiofilm activity against E. coli (BIC_50_, 1.2 μM) and to a lesser extent against B. cepacia (BIC_50_, 48.9 μM). Nifuroxazide was characterized by antibiofilm activity against E. coli (BIC_50_, 12.2 μM) and in a non-biofilm-specific way (it was active against both biofilm and planktonic cultures) against S. epidermidis (BIC_50_, 46.9 μM). Tannic acid showed antibiofilm activity against B. cepacia (BIC_50_, 1.9 μM), *S*. Typhimurium (BIC_50_, 18.8 μM), and P. aeruginosa (BIC_50_, 27.7 μM).

Furthermore, an in-house screening of more than 20,000 small molecules indicated a hit rate of 0.7% for antibiofilm compounds with BIC_50_s of ≤50 μM against *S*. Typhimurium (77), indicating that compounds with potent antibiofilm activities are rare. In addition, screening of a set of 48 in-house-developed antibiofilm compounds (with diverse scaffolds) against a subset of the biofilm assays of the current study indicated that 16 (33.3%), 2 (4.2%), 10 (20.8%), and 15 (31.3%) of these compounds had BIC_50_s of ≤50 μM against *S*. Typhimurium, P. aeruginosa (37°C), P. aeruginosa (25°C), and E. coli, respectively, whereas 11 (22.9%), 0 (0%), 9 (18.8%), and 4 (8.3%) compounds had BIC_50_s of ≤10 μM, respectively.

Other reported antibiofilm compounds generally have activities (BIC_50_s) ranging from 0.5 to 50 μM (80, 87). Moreover, Junker and Clardy performed a high-throughput screening of 66,095 small molecules against P. aeruginosa biofilms, of which only 30 compounds (0.05%) showed BIC_50_ values of ≤20 μM (81).

Hence, based on this knowledge, we classified 5-Ar-2-AIs with BIC_50_ values of *≤*50 μM as potent biofilm inhibitors and 5-Ar-2-AIs with BIC_50_ values of *≤*10 μM as very strong inhibitors.

### Preventive activity of diverse 5-Ar-2-AIs against monospecies bacterial and fungal biofilms.

**FIG 3:**
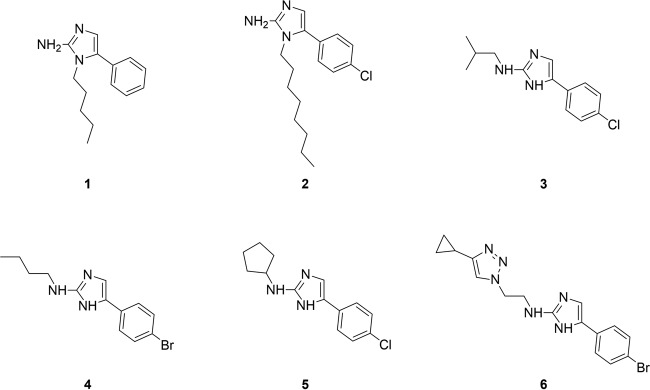
Structures of 5-Ar-2-AI-based compounds whose activities against a broad panel of monospecies and mixed-species biofilm models were tested in this study.

We selected six 5-Ar-2-AIs (Fig. 3) with previously reported potent or very strong activity against *S*. Typhimurium and P. aeruginosa (25°C) biofilms and tested their preventive antibiofilm activity against our broad panel of bacterial and fungal pathogens in a monospecies biofilm setup, by using a crystal violet-based assay and a CTB-based assay, respectively (Table 2).

**TABLE 2:**
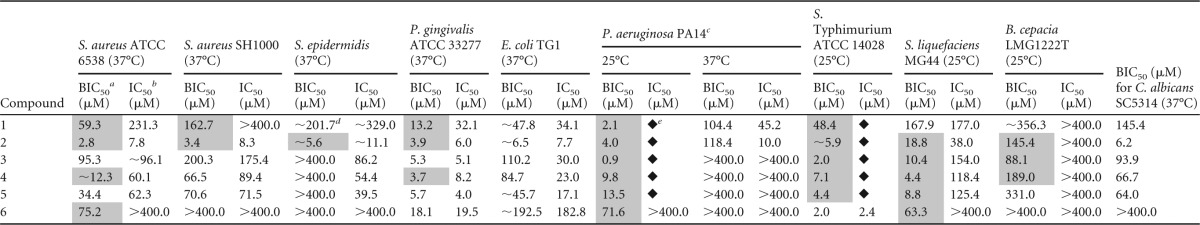
Effect of 5-Ar-2-AIs on a panel of monospecies bacterial and fungal biofilms^*f*^

a BIC_50_, concentration of compound needed to inhibit biofilm formation by 50%.

b IC_50_, concentration of compound needed to inhibit planktonic growth by 50%.

c Biofilm formation was studied at 25°C and 37°C to simulate environmental and *in vivo* conditions, respectively.

d ∼, the BIC_50_ and IC_50_ values could not be accurately calculated due to the steepness of the curve.

e ◆, the effect on planktonic growth has previously been determined by growth curve analysis (26, 27, 29).

f Results for compounds that have biofilm-specific activity (2× BIC_50_ < IC_50_) are shaded in gray. The 95% confidence intervals are provided in Table S2 in the supplemental material.

Compounds 1 and 2 are substituted at the *N*1 position of the 2-aminoimidazole moiety (26) with an alkyl group of intermediate length (Fig. 3) (26). As indicated in Table 2, compound 2 was found to be very active against the formation of biofilms by Gram-positive bacteria (S. aureus ATCC 6538, S. aureus SH1000, and S. epidermidis), with BIC_50_ values being between 2 and 6 μM. Compound 1 also had antibiofilm activity against these bacteria; however, its antibiofilm activity was more moderate. Furthermore, both compounds showed potent and specific antibiofilm activity against the Gram-negative bacteria P. gingivalis, P. aeruginosa (25°C), and *S*. Typhimurium, with BIC_50_ values being between 2 and 50 μM. Both compounds also inhibited the formation of biofilms by E. coli and P. aeruginosa at 37°C (BIC_50_ range, 6 to 120 μM); however, it was in a non-biofilm-specific way. Compound 2 but not compound 1 had potent biofilm-specific activity against S. liquefaciens biofilms (BIC_50_, 18.8 μM; IC_50_, 38.0 μM). Both compounds moderately affected the formation of biofilms by B. cepacia, with BIC_50_ values being between 145 and 400 μM. Finally, compound 2 had a very strong capacity to inhibit biofilm formation by the fungus C. albicans (BIC_50_, 6.2 μM), while compound 1 was only moderately active.

The 2*N*-substituted 2-aminoimidazoles (compounds 3 to 5) (27) in general showed only moderate, non-biofilm-specific activity against the Gram-positive bacteria S. aureus ATCC 6538 and S. aureus SH1000 (BIC_50_ range, 12.3 to 200.3 μM), while the compounds were not active against S. epidermidis. With respect to the Gram-negative bacterial species, high levels of activity were observed against P. gingivalis, P. aeruginosa (25°C), *S*. Typhimurium, and S. liquefaciens biofilms (BIC_50_ range, 1 to 15 μM), lower levels of activity were observed against E. coli and B. cepacia (BIC_50_ range, 45 to 331 μM), and no activity was observed against P. aeruginosa at 37°C. Only moderate activities against the fungus C. albicans were measured.

Finally, the 2-aminoimidazole–triazole conjugate (compound 6) (29) displayed potent, though non-biofilm-specific, activity against P. gingivalis and *S*. Typhimurium (BIC_50_s, 18.1 and 2.0 μM respectively) and moderate, biofilm-specific activity against S. aureus ATCC 6538, P. aeruginosa (25°C), and S. liquefaciens. No activity against S. aureus SH1000, S. epidermidis, P. aeruginosa (37°C), B. cepacia, and C. albicans was observed.

### Preventive activity of diverse 5-Ar-2-AIs against mixed-species bacterial biofilms.

Recent reports have indicated that mixed-species bacterial biofilms can be more resistant to antimicrobial agents than single-species biofilms (37, 46–48, 51, 52, 88, 89). The community-level resilience can, for example, be provided by one resistant species able to protect the whole community (46). Therefore, we evaluated compounds 1 to 6 (Fig. 3) for their preventive activity against a mixture of the Gram-negative bacteria E. coli and P. aeruginosa (which often co-occur in urinary tract infections) (90) and a mixture of the Gram-positive bacteria S. aureus ATCC 6538 and S. epidermidis, by using a crystal violet based assay (91).

As indicated in Table 3, all compounds tested showed potent preventive activity against both the mixture of Gram-negative bacteria and the mixture of Gram-positive bacteria, with BIC_50_ values being between 0.5 and 74.3 μM. Remarkably, the activity of the 2*N*-substituted compounds against the mixed-species biofilms was higher than that against monospecies biofilms of the constituent species.

**TABLE 3:**
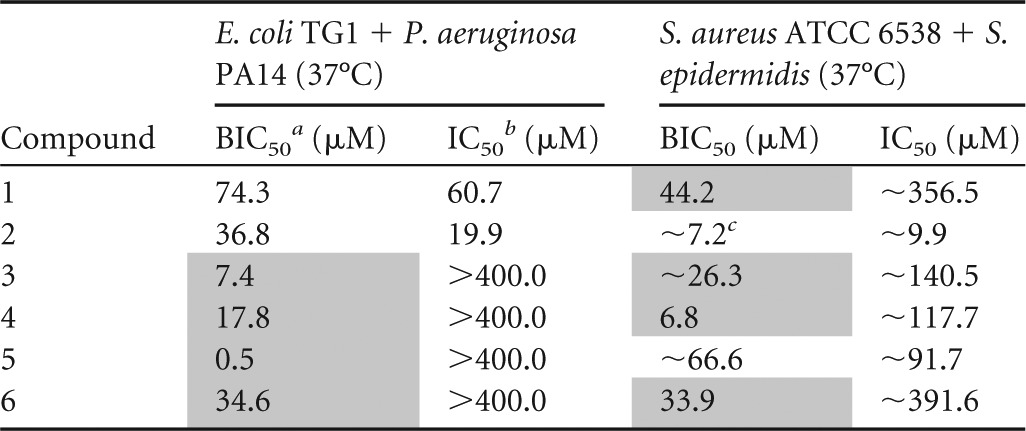
Effect of 5-Ar-2-AIs on a panel of mixed E. coli-P. aeruginosa and S. aureus-S. epidermidis biofilms^*d*^

a BIC_50_, concentration of inhibitor needed to inhibit biofilm formation by 50%.

b IC_50_, concentration of inhibitor needed to inhibit planktonic growth by 50%.

c ∼, the BIC_50_ and IC_50_ values could not be accurately calculated due to the steepness of the curve.

d Results for compounds that have biofilm-specific activity (2× BIC_50_ < IC_50_) are shaded in gray. The 95% confidence intervals are provided in Table S3 in the supplemental material.

### Preventive activity of diverse 5-Ar-2-AIs against mixed bacterial-fungal biofilms.

There is clear evidence that C. albicans interactions with bacteria play an important role in several human diseases (92, 93). An overview of bacterium-Candida interactions and their effect on fungal development is provided elsewhere (44, 45). Moreover, bacterial-fungal interactions can change the susceptibility to antimicrobial treatment (47, 54). Therefore, we evaluated compounds 2, 3, and 5 (Fig. 3) for their preventive activity against a panel of mixed bacterial-fungal biofilms, consisting of pairwise combinations of C. albicans and E. coli, S. epidermidis, and S. aureus.

As indicated in Table 4, the *N*1-substituted 5-Ar-2-AI compound 2 seems to be the compound best suited for the treatment of mixed fungal-bacterial biofilms, since at a concentration of 100 μM it caused a strong reduction of each species in the mixed biofilms tested. C. albicans-S. epidermidis biofilm formation was even completely inhibited at 25 μM.

**TABLE 4:**
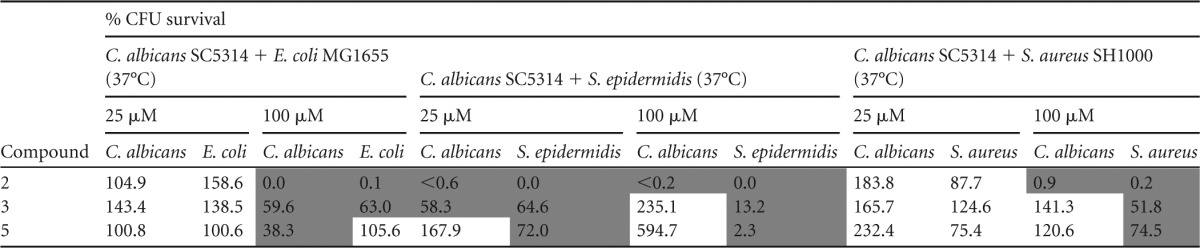
Effect of 5-Ar-2-AIs on a panel of mixed bacterial-fungal biofilms^*a*^

a Compounds with <75% CFU survival are shaded in dark gray.

The 2*N*-substituted 5-Ar-2-AI compound 3 had moderate (incomplete inhibition) activity against the C. albicans-E. coli combination, against the C. albicans-S. epidermidis combination, and against S. aureus within the C. albicans-S. aureus combination. Finally, compound 5 had strong activity (complete inhibition at 100 μM) against S. epidermidis in the C. albicans-S. epidermidis biofilm but only moderate activity against C. albicans in the C. albicans-E. coli combination and S. aureus in the C. albicans-S. aureus combination.

### Comparison of antibiofilm activity and toxicity of diverse 5-Ar-2-AIs.

Overall, it can be concluded from the results presented above that the *N*1-substituted 5-Ar-2-AI compound 2 showed the broadest activity spectrum, with strong activity against most monospecies bacterial biofilms, the monospecies C. albicans biofilm, both the mixture of Gram-negative bacteria and the mixture of Gram-positive bacteria, and all mixed bacterial-fungal biofilms. Also, the other *N*1-substituted compound, compound 1, showed activity against most of these biofilms, although at higher doses. Unfortunately, as previously reported, compound 2 and the *N*1-subsituted 5-Ar-2-AIs in general showed strong toxicity against eukaryotic tumor cell lines, bone cells, and the nematode Caenorhabditis elegans. Indeed, the *N*1-subsituted 5-Ar-2-AIs generally have a therapeutic index (TI) of less than 1 with regard to biofilm inhibition (76). TI is calculated as the ratio of the compound concentration producing toxicity against tumor cell lines (IC_50_) to the concentration needed to exert the desired therapeutic effect on biofilms (BIC_50_). The higher that the therapeutic index is, the broader that the safety window of the compound is. The 2*N*-substituted 2-aminoimidazoles compounds 3 to 5, on the other hand, had good activity against most monospecies and mixed-species biofilms of Gram-negative bacteria but had more moderate activity against the monospecies biofilms of the Gram-positive bacteria and C. albicans and against their mixed biofilms. However, the 2*N*-substituted 5-Ar-2-AIs generally have a much lower toxicity, with the TI being far greater than 1 (76). The 2-aminoimidazole–triazole conjugate (compound 6) generally has a higher level of toxicity (76) and a narrow activity spectrum against monospecies bacterial biofilms. From this analysis, it is clear that a class of nontoxic compounds with a broad spectrum of preventive activity against Gram-positive bacteria (in both monospecies and mixed-species biofilms) is currently missing. This activity profile is especially interesting for application in antibiofilm coatings for orthopedic implants, given the fact that staphylococci are most frequently associated with implant infections (13). We hypothesized that 5-Ar-2-AIs substituted at both the *N*1 and 2*N* positions might combine the broad-spectrum activity (or at least the activity against Gram-positive bacteria) of the *N*1-substituted compounds with the low toxicity of the 2*N*-substituted compounds. To test this hypothesis, a series of eight *N*1-,2*N*-disubstituted 5-Ar-2-AIs was synthesized and tested for activity against a broad panel of bacterial and fungal biofilms and for toxicity against bone cells.

### Chemical synthesis of novel compounds: *N*1-,2*N*-disubstituted 5-Ar-2-AIs.

**FIG 4:**
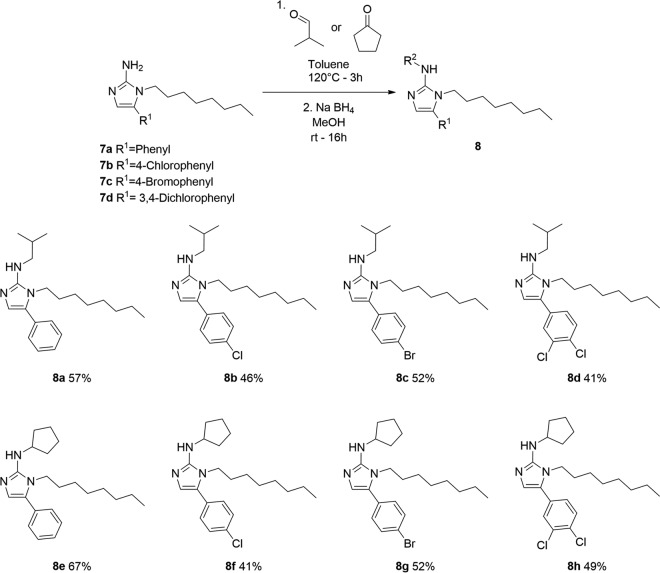
Synthesis and structures of eight novel *N*1-,2*N*-disubstituted 5-Ar-2-AIs tested against monospecies and mixed-species biofilms. MeOH, methanol; rt, room temperature. Percentages indicate compound yield.

As depicted in Fig. 4, the previously developed 2-AIs consisting of compounds 7 (26) were further functionalized by reductive amination of the 2*N* position of the 2-AIs with isobutyraldehyde and cyclopentanone. The desired *N*1-,2*N*-disubstituted 5-Ar-2-AIs consisting of compounds 8 were obtained in moderate yields. These compounds combine the *N*1-octyl substituent of compound 2 with the 2*N*-isobutyl or 2*N*-cyclopentyl substituent of compounds 3 and 5, respectively.

### Preventive activity of novel compounds against monospecies bacterial and fungal biofilms.

The preventive activity of the novel *N*1-,2*N*-disubstituted 5-Ar-2-AIs was first evaluated against a panel of monospecies bacterial and fungal biofilms. Interestingly, as indicated in Table 5, all compounds inhibited biofilm formation by the Gram-positive bacterium S. aureus ATCC 6538 (37°C) at low concentrations (BIC_50_ range, 1.0 to 41.0 μM), except for compound 8d, which had a higher BIC_50_ of 116.0 μM. Hence, these novel compounds are characterized by increased antibiofilm activity compared to that of the 5-Ar-2-AIs compounds 3 and 5, which are substituted only at the 2*N* position. Bacterial growth was not affected by these compounds at concentrations equal to the BIC_50_, except in the case of compound 8a, pointing to biofilm-specific activity.

**TABLE 5:**
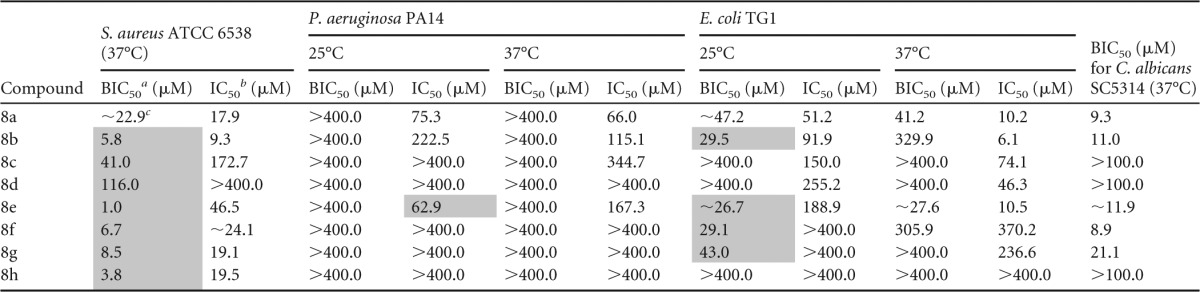
Effect of novel 5-Ar-2-AIs on a panel of monospecies biofilms of bacteria and fungi^*d*^

a BIC_50_, concentration of inhibitor needed to inhibit biofilm formation by 50%.

b IC_50_, concentration of inhibitor needed to inhibit planktonic growth by 50%.

c ∼, the BIC_50_ and IC_50_ values could not be accurately calculated due to the steepness of the curve.

d Results for compounds that have biofilm-specific activity (2× BIC_50_ < IC_50_) are shaded in gray. The 95% confidence intervals are provided in Table S4 in the supplemental material.

However, none of the compounds was active against P. aeruginosa biofilms at 25°C or 37°C, whereas the effect on E. coli biofilm formation was strongly dependent on the substitution pattern of the 5-aryl ring. Only compounds 8a, 8b, 8e, and 8f, bearing an unsubstituted phenyl ring or *para*-chlorophenyl at the 5 position of the 2-aminoimidazole ring, had potent activity against E. coli biofilm cells at 25°C, and only compounds 8a and 8e with an unsubstituted 5-phenyl ring showed activity at 37°C. The activities at 25°C were biofilm specific (except in the case of compound 8a), while at 37°C the planktonic growth was also affected. Most of the novel compounds showed a potent preventive activity against C. albicans biofilm formation, with BIC_50_ values being between 9 and 22 μM. Only compounds 8c, 8d, and 8h were not active at the highest concentration tested (100 μM). In conclusion, whereas these novel compounds had increased activity against the Gram-positive bacterium S. aureus compared to the activity of the previously described 2*N*-subsituted compounds, their activity against the Gram-negative bacteria P. aeruginosa and E. coli was reduced.

### Preventive activity of novel compounds against mixed-species biofilms.

Finally, the preventive activity of the novel *N*1-,2*N*-disubstituted 5-Ar-2-AIs was evaluated against a panel of mixed-species bacterial biofilms and mixed bacterial-fungal biofilms (Table 6). Most compounds strongly inhibited both S. epidermidis and C. albicans in the C. albicans-S. epidermidis mixture, except for compounds 8c and 8d, which reduced only C. albicans. All the novel compounds also showed a very strong, biofilm-specific effect on the S. aureus-S. epidermidis mixed biofilm, except for compound 8d. The mixed biofilm of the Gram-negative bacteria P. aeruginosa and E. coli, on the other hand, was strongly inhibited only by compound 8a and at higher concentrations by compounds 8e and 8f. In agreement with the results of the monospecies biofilm assays, these novel compounds generally showed very strong activity against the Gram-positive bacteria and C. albicans in the mixed biofilms; however, they had only poor activity against the Gram-negative bacteria.

**TABLE 6:**
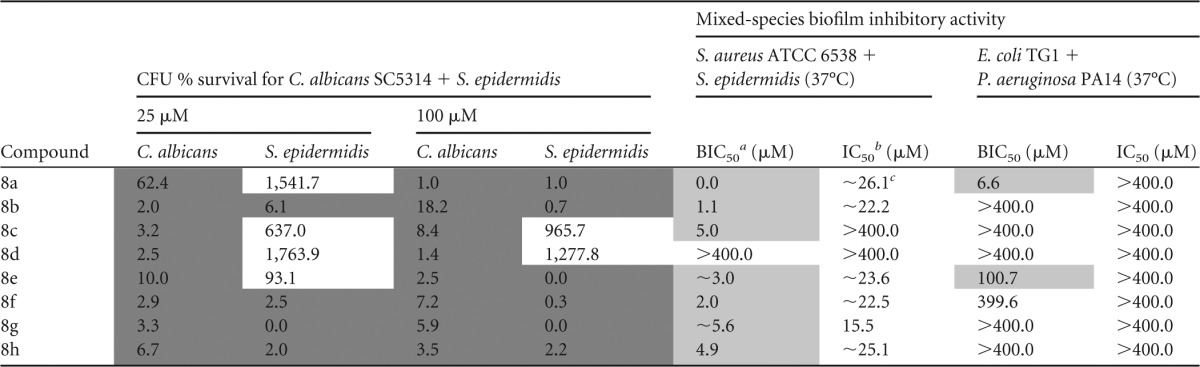
Effect of novel 5-Ar-2-AIs on a panel of mixed species biofilms^*d*^

a BIC_50_, concentration of inhibitor needed to inhibit biofilm formation by 50%.

b IC_50_, concentration of inhibitor needed to inhibit planktonic growth by 50%.

c ∼, the BIC_50_ and IC_50_ values could not be accurately calculated due to the steepness of the curve.

d Results for compounds that have biofilm-specific activity (2× BIC_50_ < IC_50_) are shaded in light gray, and results for compounds with <75% CFU survival are shaded in dark gray. The 95% confidence intervals are provided in Table S5 in the supplemental material.

### Effect of novel compounds on viability and functional behavior of bone cells.

The novel compounds have an interesting activity profile for application in antibiofilm coatings for orthopedic implants. Moreover, preliminary experiments indicated that these compounds retain their activity after covalent attachment to a surface, making them suitable for incorporation in both covalent antibiofilm coatings and slow-release coatings. In light of the application of these compounds as anti-infective coatings on orthopedic implants, we determined their effect on the viability and functional behavior of bone cells. Additionally, this allowed an easy comparison with the toxicity of the previously described 5-Ar-2-AIs, which was evaluated using the same assays described here (76).

**FIG 5:**
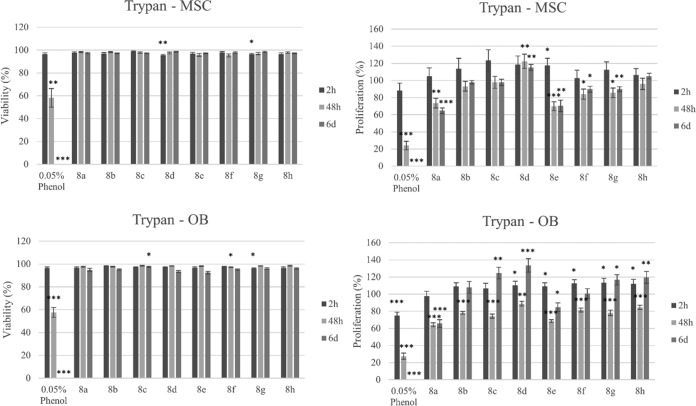
Effects of selected compounds (12.5 μM) on the proliferation and viability of OB and MSC after 2 h, 48 h, and 6 days (6d) of exposure, as determined by trypan blue staining. Bars and error bars represent the means and standard errors from eight repeats, respectively. The negative control was cell culture medium with a 0.5% ethanol solvent background, and the positive control was 0.05% phenol to show a cytotoxic effect. Percent proliferation is defined as (total number of viable cells in treated sample/total number of viable cells in solvent control) × 100. Percent viability is defined as (total number of viable cells [unstained]/total number of cells [stained and unstained]) × 100. Significant differences (*, *P* < 0.05; **, *P* < 0.01; ***, *P* < 0.001) from the results for the negative control are indicated.

**FIG 6:**
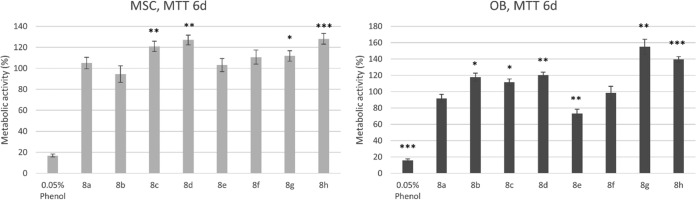
Effect of selected compounds (12.5 μM) on the metabolic activity of OB and MSC after 6 days of exposure, as determined by MTT staining. Bars and error bars represent the means and standard errors from four repeats, respectively. The negative control was cell culture medium with 0.5% ethanol solvent background, and the positive control was 0.05% phenol to show a cytotoxic effect. Significant differences (*, *P* < 0.05; **, *P* < 0.01; ***, *P* < 0.001) from the results for the negative control are indicated.

The effect of the novel compounds on the viability (i.e., the percentage of viable cells in treated sample compared to the total number [viable and nonviable] of cells in the treated sample) of osteoblasts (OB) and mesenchymal stem cells (MSC) as a function of time was first tested. For each compound, a dose of 12.5 μM, which is well above the BIC_50_ value of most compounds for S. aureus and S. aureus-S. epidermidis biofilm inhibition, was used. As shown in Fig. 5, cell viability, measured by trypan blue staining, was only very slightly reduced (<10%) early in the treatment with a limited number of compounds. After 6 days of exposure, none of the compounds altered the viability of the two cell types, except for compound 8c, which very slightly reduced the viability of OB. MTT staining indicated that the metabolic activity of both cell types was even increased compared to that of the solvent control after 6 days of treatment with compounds 8c, 8d, 8g, and 8h (Fig. 6), all of which bore a *para*-bromophenyl or 3,4-dichlorophenyl substituent at the 5 position of the imidazole ring. Interestingly, an increase in proliferation (Fig. 5) was also observed after 6 days of exposure to compounds 8c, 8d, 8g, and 8h (OB) and compound 8d (MSC). The proliferation of MSC and OB was, however, slightly reduced after 6 days treatment with compounds 8a, 8e, 8f, and 8g and with compounds 8a and 8e, respectively.

**FIG 7:**
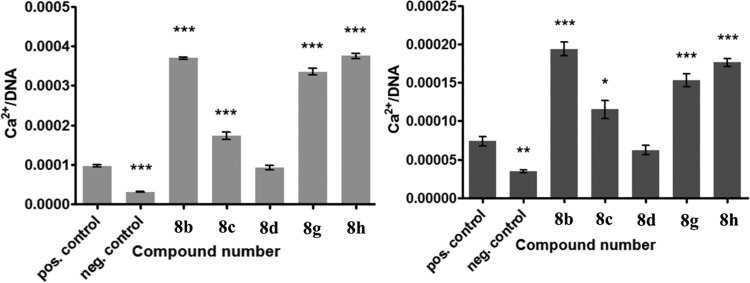
Effect of selected compounds (12.5 μM) on the osteogenic differentiation potential of MSC (left) and OB (right) after 5 and 3 weeks of exposure, respectively, as determined by measuring the calcium content, which was normalized by the amount of DNA. Bars and error bars represent the means and standard errors from at least four repeats, respectively. The negative control contains no osteogenic supplements. The solvent (positive) control contains osteogenic supplements and a 0.5% ethanol background. Significant differences (*, *P* < 0.05; **, *P* < 0.01; ***, *P* < 0.001) from the results for the solvent control are indicated. pos., positive; neg., negative.

Next, compounds 8b, 8c, 8d, 8g, and 8h, which allowed survival of MSC and OB for more than 3 weeks, were tested for their osteogenic differentiation potential, as those two cell types are responsible for the production of new bone matrix within bone tissue. Calcium deposition was chosen as an indicator of the osteogenic phenotype, as it is the final and functional marker of osteoblast differentiation. As shown in Fig. 7, none of the compounds at 12.5 μM negatively affected the calcium deposition of either of the two cell types. Interestingly, all compounds except compound 8d significantly (*P* < 0.05 for compound 8c with OB, *P* < 0.001 for the rest of the compounds) induced the calcium deposition of both cell types. This indicates that antibiofilm coating of orthopedic implants with these compounds might even stimulate the osseointegrative potential.

### Conclusions.

In the present study, we evaluated the activities of a selection of our previously reported 5-aryl-2-aminoimidazoles (5-Ar-2-AIs) (Fig. 3) against a broad panel of monospecies and mixed-species biofilm models. The *N*1-substituted 5-Ar-2-AI compound 2 showed the broadest activity spectrum, with very strong activity against Gram-negative and Gram-positive bacteria and the fungus C. albicans both in monospecies and in mixed-species biofilm models. Unfortunately, this compound and *N*1-substituted 5-Ar-2-AIs in general have high levels of toxicity against eukaryotic tumor cell lines, bone cells, and the nematode Caenorhabditis elegans (76). The 2*N*-substituted 2-aminoimidazoles compounds 3 to 5, on the other hand, are not toxic (76) and showed good activity against most monospecies and mixed-species biofilms of Gram-negative bacteria, but in general, they had only moderate activity against the biofilms formed by monospecies of Gram-positive bacteria and C. albicans as well as their mixed biofilms. The 2-aminoimidazole–triazole conjugate compound 6 had a higher level of toxicity (76) and a narrow spectrum of activity against monospecies bacterial biofilms. In an attempt to develop nontoxic compounds with broad activity at least against Gram-positive bacteria in monospecies and mixed-species biofilms, we synthesized a series of eight novel 5-Ar-2-AIs with substituents at both the *N*1 and 2*N* positions (Fig. 4). This activity profile is especially interesting for application in antibiofilm coatings for medical implants, such as orthopedic prostheses, given the fact that staphylococci are most frequently associated with implant infections. As desired, most of these novel compounds showed very strong activity against the Gram-positive bacteria (S. aureus and S. epidermidis) and C. albicans in all monospecies and mixed-species biofilms tested, albeit at the cost of a loss of activity against the Gram-negative species P. aeruginosa and E. coli. None of the novel compounds strongly affected the viability or proliferation of osteoblasts and bone marrow-derived stem cells, and remarkably, most of the compounds even induced the calcium deposition of both cell types, suggesting that an antibiofilm coating of orthopedic implants with these compounds might even stimulate the osseointegrative potential. In conclusion, our data show that modulation of the substitution pattern of the 5-Ar-2-AI scaffold allows fine-tuning of both the antibiofilm activity spectrum and toxicity.

## Supplementary Material

Supplemental material
