# Supplementary material for: Modulation of the Substitution Pattern of 5-Aryl-2-Aminoimidazoles Allows Fine-Tuning of Their Antibiofilm Activity Spectrum and Toxicity
Source: Antimicrob Agents Chemother. 2016 Oct 21;60(11):6483–97. doi: 10.1128/AAC.00035-16 (PMC5075052; doi:10.1128/AAC.00035-16)
Supplement: Supplemental material [file supp_60_11_6483__index.html]

Supplemental material 

# Modulation of the Substitution Pattern of 5-Aryl-2-Aminoimidazoles Allows Fine-Tuning of Their Antibiofilm Activity Spectrum and Toxicity

## Supplemental material

- Supplemental file 1 -

  Supplemental text and Tables S1-S5

  PDF, 202K
