# Supplementary material for: Modulation of the Substitution Pattern of 5-Aryl-2-Aminoimidazoles Allows Fine-Tuning of Their Antibiofilm Activity Spectrum and Toxicity
Source: Antimicrob Agents Chemother. 2016 Oct 21;60(11):6483–97. doi: 10.1128/AAC.00035-16 (PMC5075052; doi:10.1128/AAC.00035-16)
Supplement: Supplemental material [file AAC.00035-16_zac011165629so1.pdf]

NMR spectra

N-isobutyl-1-octyl-5-phenyl-1H-imidazol-2-amine (**8a**). Obtained from the general procedure as an orange oil, yield 57%. ^{1}H NMR (300 MHz, Chloroform-d) δ 7.52 – 7.20 (m, 5H), 6.71 (s, 1H), 3.71 (t, *J* = 7.5 Hz, 3H), 3.23 (t, *J* = 6.4 Hz, 2H), 1.96 (dp, *J* = 13.4, 6.7 Hz, 1H), 1.80 – 1.45 (m, 2H), 1.18 (s, 10H), 1.00 (d, *J* = 6.6 Hz, 6H), 0.86 (t, *J* = 6.8 Hz, 3H). ^{13}C NMR (75 MHz, $CDCl_3$) δ 150.75, 131.13, 129.25, 128.67, 128.60, 128.23, 128.16, 127.05, 123.00, 51.56, 42.64, 31.67, 29.42, 29.02, 28.98, 28.94, 28.42, 26.51, 22.58, 20.32, 14.06.

5-(4-chlorophenyl)-N-isobutyl-1-octyl-1H-imidazol-2-amine (**8b**). Obtained from the general procedure as an orange oil, yield 46%. ^{1}H NMR (300 MHz, Chloroform-d) δ 7.37 (d, *J* = 8.1 Hz, 2H), 7.17 (d, *J* = 8.2 Hz, 2H), 6.69 (s, 1H), 3.79 (t, *J* = 7.5 Hz, 2H), 3.16 (d, *J* = 6.9 Hz, 2H), 1.98 – 1.81 (m, 1H), 1.48 (t, *J* = 7.5 Hz, 2H), 1.32 – 1.03 (m, 10H), 0.95 (d, *J* = 6.6 Hz, 6H), 0.85 (t, *J* = 6.9 Hz, 3H). ^{13}C NMR (75 MHz, CDCl3) δ 148.89, 134.47, 129.77, 129.11, 127.79, 127.32, 115.99, 51.07, 43.09, 31.61, 28.93, 28.84, 28.80, 28.50, 28.27, 26.15, 22.54, 22.52, 19.99, 14.02.

5-(4-bromophenyl)-N-isobutyl-1-octyl-1H-imidazol-2-amine (**8c**). Obtained from the general procedure as a yellow oil, yield 52%. ^{1}H NMR (300 MHz, Chloroform-d) δ 7.62 – 7.45 (m, 2H), 7.25 – 7.10 (m, 2H), 6.72 (s, 1H), 3.79 – 3.57 (m, 3H), 3.23 (t, *J* = 6.3 Hz, 2H), 1.95 (dt, *J* = 13.4, 6.7 Hz, 1H), 1.57 (t, *J* = 7.5 Hz, 2H), 1.32 – 1.11 (m, 10H), 1.00 (d, *J* = 6.6 Hz, 6H), 0.87 (t, *J* = 6.8 Hz, 3H). ^{13}C NMR (75 MHz, $CDCl_3$) δ 151.09, 131.78, 130.10, 129.54, 128.01, 123.68, 120.94, 51.50, 42.68, 31.68, 29.44, 29.03, 28.95, 28.41, 26.50, 22.60, 20.30, 14.07.

5-(3,4-dichlorophenyl)-N-isobutyl-1-octyl-1H-imidazol-2-amine (**8d**). Obtained from the general procedure as an orange oil, yield 41%. ^{1}H NMR (300 MHz, Chloroform-d) δ 7.43 – 7.28 (m, 2H), 7.06 (dt, *J* = 8.3, 1.9 Hz, 1H), 6.56 (d, *J* = 29.1 Hz, 1H), 4.12 – 3.53 (m, 3H),

3.54 – 2.88 (m, 1H), 1.78 – 1.47 (m, 2H), 1.29 – 0.96 (m, 12H), 0.96 – 0.86 (m, 4H), 0.86 – 0.64 (m, 4H). ^{13}C NMR (75 MHz, $CDCl_3$) δ 151.41, 131.54, 130.64, 129.64, 129.11, 125.61, 124.79, 122.55, 41.74, 39.10, 30.63, 28.52, 28.12, 26.49, 25.61, 25.50, 22.86, 22.66, 21.56, 19.94, 16.61, 13.02.

N-cyclopentyl-1-octyl-5-phenyl-1H-imidazol-2-amine (**8e**). Obtained from the general procedure as an orange oil, yield 67%. ^{1}H NMR (300 MHz, Chloroform-d) δ 7.32 (tt, *J* = 12.4, 7.3 Hz, 5H), 6.73 (s, 1H), 4.20 (q, *J* = 6.3 Hz, 1H), 3.69 (t, *J* = 7.6 Hz, 2H), 3.53 (d, *J* = 6.1 Hz, 1H), 2.10 (tt, *J* = 12.2, 5.0 Hz, 2H), 1.91 – 1.61 (m, 3H), 1.53 (dq, *J* = 12.1, 6.3, 5.7 Hz, 4H), 1.39 – 1.05 (m, 10H), 0.86 (t, *J* = 6.8 Hz, 3H). ^{13}C NMR (75 MHz, $CDCl_3$) δ 150.40, 131.21, 129.06, 128.52, 128.08, 128.06, 126.90, 126.88, 123.28, 55.21, 42.51, 35.46, 33.71, 33.69, 31.63, 29.32, 28.96, 28.86, 26.37, 26.35, 23.73, 23.32, 22.55.

5-(4-chlorophenyl)-N-cyclopentyl-1-octyl-1H-imidazol-2-amine (**8f**). Obtained from the general procedure as an orange oil, yield 41%. ^{1}H NMR (300 MHz, Chloroform-d) δ 7.49 – 7.30 (m, 2H), 7.26 (d, *J* = 3.9 Hz, 2H), 6.73 (s, 1H), 4.19 (q, *J* = 6.2 Hz, 1H), 3.66 (t, *J* = 7.6 Hz, 2H), 3.44 (d, *J* = 6.2 Hz, 1H), 2.26 – 2.00 (m, 2H), 1.79 – 1.61 (m, 2H), 1.52 (ddd, *J* = 12.6, 7.9, 5.4 Hz, 4H), 1.34 – 1.07 (m, 12H), 0.87 (t, *J* = 6.8 Hz, 3H). ^{13}C NMR (75 MHz, $CDCl_3$) δ 129.46, 128.93, 127.98, 55.40, 42.80, 33.72, 31.68, 29.25, 29.02, 28.90, 26.40, 23.76, 22.60, 14.07.

5-(4-bromoophenyl)-N-cyclopentyl-1-octyl-1H-imidazol-2-amine (**8g**). Obtained from the general procedure as an orange oil, yield 52%. ^{1}H NMR (300 MHz, Chloroform-d) δ 7.52 (dd, *J* = 8.5, 2.0 Hz, 2H), 7.20 (dd, *J* = 8.4, 2.0 Hz, 2H), 6.75 (d, *J* = 2.1 Hz, 1H), 4.20 (q, *J* = 6.3 Hz, 1H), 3.67 (t, *J* = 7.5 Hz, 2H), 3.44 (d, *J* = 6.2 Hz, 1H), 2.27 – 1.98 (m, 2H), 1.94 – 1.62 (m, 4H), 1.53 (dq, *J* = 11.6, 5.7 Hz, 4H), 1.19 (s, 10H), 0.88 (t, *J* = 6.5 Hz, 3H). ^{13}C NMR (75 MHz, $CDCl_3$) δ 131.79, 130.13, 129.56, 127.96, 123.82, 120.96, 55.29, 42.66, 33.81, 31.69, 29.42, 29.03, 28.92, 26.46, 23.75, 22.61, 14.08.

5-(3,4-dichlorophenyl)-N-cyclopentyl-1-octyl-1H-imidazol-2-amine (**8h**). Obtained from the general procedure as a yellow oil, yield 49%. ^{1}H NMR (300 MHz, Chloroform-d) δ 7.49 – 7.36 (m, 2H), 7.14 (dd, *J* = 8.3, 2.1 Hz, 1H), 6.76 (s, 1H), 4.19 (q, *J* = 6.3 Hz, 1H), 3.80 – 3.57 (m, 2H), 3.50 (d, *J* = 6.2 Hz, 1H), 2.22 – 1.98 (m, 2H), 1.80 – 1.61 (m, 4H), 1.60 – 1.43 (m, 4H), 1.19 (d, *J* = 3.1 Hz, 10H), 0.87 (t, *J* = 6.8 Hz, 3H). ^{13}C NMR (75 MHz, $CDCl_3$) δ 151.06, 132.70, 131.31, 130.75, 130.58, 129.29, 126.93, 126.78, 124.82, 55.27, 42.74, 33.79, 31.68, 29.42, 29.04, 28.91, 26.43, 23.74, 22.60, 14.07.

TABLE S1 Effect of literature compounds on a panel of monospecies biofilms

	S. aureus ATCC6538				*S. aureus* SH1000				*S. epidermidis*				*E. coli* TG1				*P. aeruginosa* PA14			
	37 °C				37 °C				37 °C				37 °C				25 °C			
compd	BIC_{50} [a] (µM)	95% confidence interval for BIC_{50}	IC_{50} [b] (µM)	95% confidence interval for IC_{50}	BIC_{50} (µM)	95% confidence interval for BIC_{50}	IC_{50} (µM)	95% confidence interval for IC_{50}	BIC_{50} (µM)	95% confidence interval for BIC_{50}	IC_{50} (µM)	95% confidence interval for IC_{50}	BIC_{50} (µM)	95% confidence interval for BIC_{50}	IC_{50} (µM)	95% confidence interval for IC_{50}	BIC_{50} (µM)	95% confidence interval for BIC_{50}	IC_{50} (µM)	95% confidence interval for IC_{50}
**baicalein**	> 400.0		> 400.0		77.5	64.1 - 93.6	> 400.0		> 400.0		> 400.0		1.2	1.0 - 1.4	~ 23.5		> 400.0		> 400.0	
**nifuroxazide**	65.5	51.5 - 83.3	125.4	112.3 - 140.0	> 400.0		> 400.0		46.9	29.7 - 74.2	~ 43.4 [c]		12.2	9.7 - 15.5	> 400.0		> 400.0		> 400.0	
**tannic acid**	~ 300.0		> 400.0		115.3	97.1 - 137.0	> 400.0		> 400.0		> 400.0		~ 195.0		> 400.0		27.7	17.8 - 43.1	> 400.0	

	S. Typhimurium ATCC14028				*S. liquefaciens* MG44				*B. cepacia* LMG1222T				*C. albicans* SC5314	
	25 °C				25 °C				25 °C				37 °C	
compd	BIC_{50} (µM)	95% confidence interval for BIC_{50}	IC_{50} (µM)	95% confidence interval for IC_{50}	BIC_{50} (µM)	95% confidence interval for BIC_{50}	IC_{50} (µM)	95% confidence interval for IC_{50}	BIC_{50} (µM)	95% confidence interval for BIC_{50}	IC_{50} (µM)	95% confidence interval for IC_{50}	BIC_{50} (µM)	95% confidence interval for BIC_{50}
**baicalein**	> 400.0		> 400.0		> 400.0		> 400.0		48.9	31.2 - 76.7	> 400.0		272.9	243.9 - 305.4
**nifuroxazide**	> 400.0		> 400.0		> 400.0		> 400.0		> 400.0		> 400.0		> 400.0	
**tannic acid**	18.8	12.2 - 29.0	> 400.0		> 400.0		> 400.0		1.9	1.1 - 3.2	> 400.0		> 400.0	

[a] BIC_{50}: concentration of compound needed to inhibit biofilm formation by 50%. [b] IC_{50}: concentration of compound needed to inhibit planktonic growth by 50%. [c] ~: The $B(IC)_{50}$ values could not be accurately calculated due to the steepness of the curve. Compounds that have a biofilm-specific activity ($2 \times BIC_{50} < IC_{50}$) are marked in grey.

TABLE S2 Effect of 5-Ar-2AIs on a panel of monospecies bacterial and fungal biofilms

	S. aureus ATCC6538				*S. aureus* SH1000				*S. epidermidis*				*P. gingivalis* ATCC33277				*E. coli* TG1			
	37 °C				37 °C				37 °C				37 °C				37 °C			
compd	BIC_{50} [a] (µM)	95% confidence interval for BIC_{50}	IC_{50} [b] (µM)	95% confidence interval for IC_{50}	BIC_{50} (µM)	95% confidence interval for BIC_{50}	IC_{50} (µM)	95% confidence interval for IC_{50}	BIC_{50} (µM)	95% confidence interval for BIC_{50}	IC_{50} (µM)	95% confidence interval for IC_{50}	BIC_{50} (µM)	95% confidence interval for BIC_{50}	IC_{50} (µM)	95% confidence interval for IC_{50}	BIC_{50} (µM)	95% confidence interval for BIC_{50}	IC_{50} (µM)	95% confidence interval for IC_{50}
**1**	59.3	25.1 - 140.3	231.3	220.0 - 243.2	162.7	112.1 - 236.0	> 400.0		~ 201.7 [d]		~ 329.0		13.2	9.0 - 19.2	32.1	12.3 - 83.2	~ 47.8		34.1	29.4 - 39.6
**2**	2.8	1.9 - 4.0	7.8	3.8 - 16.0	3.4	2.1 - 5.4	8.3	6.2 - 11.1	~ 5.6		~ 11.1		3.9	3.2 - 4.6	6.0	2.3 - 15.3	~ 6.5		7.7	5.7 - 10.4
**3**	95.3	41.4 - 219.5	~ 96.1		~ 200.3		~ 175.4		> 400.0		86.2	63.1 - 117.8	5.3	2.5 - 11.3	5.1	3.1 - 8.4	110.2	73.8 - 164.6	30.0	24.3 - 37.0
**4**	~ 12.3		60.1	50.0 - 72.3	66.5	49.1 - 90.0	89.4	75.5 - 105.8	> 400.0		54.4	38.0 - 77.7	3.7	2.0 - 6.8	8.2	5.4 - 12.3	84.7	45.7 - 157.0	23.0	15.7 - 33.7
**5**	34.4	22.2 - 53.3	62.3	52.1 - 74.6	70.6	51.3 - 97.1	71.5	51.8 - 98.7	> 400.0		39.5	25.4 - 61.5	5.7	2.6 - 12.6	4.0	1.4 - 11.7	~ 45.7		17.1	14.3 - 20.5
**6**	75.2	41.4 - 136.6	> 400.0		> 400.0		> 400.0		> 400.0		> 400.0		18.1	7.7 - 42.5	19.5	7.7 - 49.2	~ 192.5		182.8	108.1 - 309.3

	P. aeruginosa PA14								*S.* Typhimurium ATCC14028				*S. liquefaciens* MG44				*B. cepacia* LMG1222T				*C. albicans* SC5314	
	25 °C [c]				37 °C [c]				25 °C				25 °C				25 °C				37 °C	
compd	BIC_{50} (µM)	95% confidence interval for BIC_{50}	IC_{50} (µM)	95% confidence interval for IC_{50}	BIC_{50} (µM)	95% confidence interval for BIC_{50}	IC_{50} (µM)	95% confidence interval for IC_{50}	BIC_{50} (µM)	95% confidence interval for BIC_{50}	IC_{50} (µM)	95% confidence interval for IC_{50}	BIC_{50} (µM)	95% confidence interval for BIC_{50}	IC_{50} (µM)	95% confidence interval for IC_{50}	BIC_{50} (µM)	95% confidence interval for BIC_{50}	IC_{50} (µM)	95% confidence interval for IC_{50}	BIC_{50} (µM)	95% confidence interval for BIC_{50}
**1**	2.1	1.2 - 3.7	♦		104.4	55.8 - 195.3	45.2	36.3 - 56.1	48.4	37.5 - 62.4	♦		167.9	115.1 - 244.8	177.0	127.3 - 246.2	~ 356.3		> 400.0		145.4	130.8 – 161.6
**2**	4.0	3.0 - 5.2	♦		118.4	57.8 - 242.5	10.0	8.0 - 12.6	~ 5.9		♦		18.8	14.2 - 24.9	38.0	26.8 - 54.0	145.4	9.5 - 400.0	> 400.0		6.2	5.1 – 7.4
**3**	0.9	0.5 - 1.8	♦		> 400.0		> 400.0		2.0	1.6 - 2.5	♦		10.4	8.7 - 12.6	154.0	116.4 - 203.8	88.1	21.5 - 360.9	> 400.0		93.9	80.1 – 110.2
**4**	9.8	6.8 - 14.1	♦		> 400.0		> 400.0		7.1	3.7 - 13.9	♦		4.4	3.7 - 5.3	118.4	88.0 - 159.3	189.0	56.6 - 400.0	> 400.0		66.7	56.0 – 79.4
**5**	13.5	9.0 - 20.5	♦		> 400.0		> 400.0		4.4	4.0 - 4.8	♦		8.8	6.7 - 11.7	125.4	92.8 - 169.3	331.0	84.4 - 400.0	> 400.0		64.0	57.2 – 71.8
**6**	71.6	21.8 - 234.8	> 400.0		> 400.0		> 400.0		2.0	1.4 - 2.9	2.4	0.9 - 6.3	63.3	16.2 - 246.9	> 400.0		> 400.0		> 400.0		> 400.0	

[a] BIC_{50}: concentration of compound needed to inhibit biofilm formation by 50%. [b] IC_{50}: concentration of compound needed to inhibit planktonic growth by 50%. [c] Biofilm formation was studied at 25 °C and 37 °C to simulate environmental and in vivo conditions, respectively. [d] ~: The $B(IC)_{50}$ values could not be accurately calculated due to the steepness of the curve. ♦ The effect on the planktonic growth has previously been determined by growth curve analysis (1-3). Compounds that have a biofilm-specific activity (2 x BIC_{50} < IC_{50}) are marked in grey.

TABLE S3 Effect of 5-Ar-2AIs on a panel of mixed *E. coli/P. aeruginosa* and *S. aureus/S. epidermidis* biofilms

	E. coli TG1 + *P. aeruginosa* PA14				*S. aureus* ATCC6538 + *S. epidermidis*			
	37 °C				37 °C			
compd	BIC_{50} [a] (µM)	95% confidence interval for BIC_{50}	IC_{50} [b] (µM)	95% confidence interval for IC_{50}	BIC_{50} (µM)	95% confidence interval for BIC_{50}	IC_{50} (µM)	95% confidence interval for IC_{50}
1	74.3	33.0 – 167.7	60.7	42.4 – 87.1	44.2	18.4 – 119.0	~ 356.5 [c]	
2	36.8	16.2 – 85.0	19.9	14.6 – 26.9	~ 7.2		~ 9.9	
3	7.4	3.8 – 21.5	> 400.0		~ 26.3		~ 140.5	
4	17.8	12.7 – 58.2	> 400.0		6.8	1.7 – 36.3	~ 117.7	
5	0.5	0.2 – 1.5	> 400.0		~ 66.6		~ 91.7	
6	34.6	17.9 – 68.5	> 400.0		33.9	17.1 – 67.5	~ 391.6	

[a] BIC_{50}: concentration of inhibitor needed to inhibit biofilm formation by 50%. [b] IC_{50}: concentration of inhibitor needed to inhibit planktonic growth by 50%. [c] ~: The $B(IC)_{50}$ values could not be accurately calculated due to the steepness of the curve. Compounds that have a biofilm-specific activity (2 x BIC_{50} < IC_{50}) are marked in grey.

TABLE S4 Effect of novel 5-Ar-2AIs on a panel of monospecies biofilms of bacteria and fungi.

	S. aureus ATCC6538				*P. aeruginosa* PA14								*E. coli* TG1								*C. albicans* SC5314	
	37 °C				25 °C				37 °C				25 °C				37 °C				37 °C	
compd	BIC_{50} [a] (µM)	95% confidence interval for BIC_{50}	IC_{50} [b] (µM)	95% confidence interval for IC_{50}	BIC_{50} (µM)	95% confidence interval for BIC_{50}	IC_{50} (µM)	95% confidence interval for IC_{50}	BIC_{50} (µM)	95% confidence interval for BIC_{50}	IC_{50} (µM)	95% confidence interval for IC_{50}	BIC_{50} (µM)	95% confidence interval for BIC_{50}	IC_{50} (µM)	95% confidence interval for IC_{50}	BIC_{50} (µM)	95% confidence interval for BIC_{50}	IC_{50} (µM)	95% confidence interval for IC_{50}	BIC_{50} (µM)	95% confidence interval for BIC_{50}
**8a**	~ 22.9 [c]		17.9	15.3 – 21.0	> 400.0		75.3	46.9 - 121.1	> 400.0		66.0	46.9 - 92.8	~ 47.2		51.2	29.1 - 90.2	41.2	25.3 – 67.0	10.2	4.4 – 23.9	9.3	7.5 – 11.7
**8b**	5.8	2.5 – 13.5	9.3	8.3 - 10.3	> 400.0		222.5	143.6 - 344.8	> 400.0		115.1	89.0 – 149.0	29.5	15.7 - 55.4	91.9	33.5 - 252.1	329.9	161.9 – 400.0	6.1	3.5 - 10.8	11.0	8.2 – 14.7
**8c**	41.0	2.1 – 400.0	172.7	151.1 - 197.3	> 400.0		> 400.0		> 400.0		344.7	282.2 – 400.0	> 400.0		150.0	84.8 - 265.2	> 400.0		74.1	21.8 - 252.4	> 100.0	
**8d**	116.0	21.6 – 400.0	> 400.0		> 400.0		> 400.0		> 400.0		> 400.0		> 400.0		255.2	194.1 - 335.5	> 400.0		46.3	25.8 - 83.2	> 100.0	
**8e**	1.0	0.5 - 1.7	46.5	24.1 – 89.8	> 400.0		62.9	47.9 – 82.5	> 400.0		167.3	110.8 - 252.6	~ 26.7		188.9	105.7 - 337.5	~ 27.6		10.5	6.2 - 17.7	~ 11.9	
**8f**	6.7	3.2 - 13.8	~ 24.1		> 400.0		> 400.0		> 400.0		> 400.0		29.1	18.1 - 46.9	> 400.0		305.9	94.2 – 400.0	370.2	39.2 – 400.0	8.9	7.1 – 11.2
**8g**	8.5	3.3 – 22.0	19.1	16.1 - 22.6	> 400.0		> 400.0		> 400.0		> 400.0		43.0	10.8 - 170.7	> 400.0		> 400.0		236.6	108.4 – 400.0	21.1	16.2 – 27.4
**8h**	3.8	1.5 - 9.8	19.5	14.4 - 26.6	> 400.0		> 400.0		> 400.0		> 400.0		> 400.0		> 400.0		> 400.0		> 400.0		> 100.0	

[a] BIC_{50}: concentration of inhibitor needed to inhibit biofilm formation by 50%. [b] IC_{50}: concentration of inhibitor needed to inhibit planktonic growth by 50%. [c] ~: The $B(IC)_{50}$ values could not be accurately calculated due to the steepness of the curve. Compounds that have a biofilm-specific activity (2 x BIC_{50} < IC_{50}) are marked in grey.

TABLE S5 Effect of novel 5-Ar-2AIs on a panel of mixed species biofilms

	C. albicans SC5314 + *S. epidermidis*				*S. aureus* ATCC6538 + *S. epidermidis*				*E. coli* TG1 + *P. aeruginosa* PA14			
	CFUs % survival				37 °C				37 °C			
	25 µM		100 µM									
compd	*C.a.*	*S.e.*	*C.a.*	*S.e.*	BIC_{50} (µM)[a]	95% confidence interval for BIC_{50}	IC_{50} (µM)[b]	95% confidence interval for IC_{50}	BIC_{50} (µM)	95% confidence interval for BIC_{50}	IC_{50} (µM)	95% confidence interval for IC_{50}
**8a**	62.4	1541.7	1.0	1.0	0.0	0.0 – 0.3	~ 26.1[c]		6.6	2.3 – 19.2	> 400.0	
**8b**	2.0	6.1	18.2	0.7	1.1	0.7 – 1.7	~ 22.2		> 400.0		> 400.0	
**8c**	3.2	637.0	8.4	965.7	5.0	2.9 – 8.5	> 400.0		> 400.0		> 400.0	
**8d**	2.5	1763.9	1.4	1277.8	> 400.0		> 400.0		> 400.0		> 400.0	
**8e**	10.0	93.1	2.5	0.0	~ 3.0		~ 23.6		100.7	7.5 – 400.0	> 400.0	
**8f**	2.9	2.5	7.2	0.3	2.0	1.1 – 3.5	~ 22.5		399.6	26.9 – 400.0	> 400.0	
**8g**	3.3	0.0	5.9	0.0	~ 5.6		15.5	9.9 – 24.4	> 400.0		> 400.0	
**8h**	6.7	2.0	3.5	2.2	4.9	2.9 – 8.2	~ 25.1		> 400.0		> 400.0	

C.a. : *Candida albicans*; *S.e.* : *Staphylococcus epidermidis.* [a] BIC_{50}: concentration of inhibitor needed to inhibit biofilm formation by 50%. [b] IC_{50}: concentration of inhibitor needed to inhibit planktonic growth by 50%. [c] ~: The $B(IC)_{50}$ values could not be accurately calculated due to the steepness of the curve. Compounds that have a biofilm-specific activity (2 x BIC_{50} < IC_{50}) are marked in grey and compounds with < 75 % CFU survival are marked in dark grey.

REFERENCES

1. **Steenackers HP, Ermolat'ev DS, Savaliya B, De Weerdt A, De Coster D, Shah A, Van der Eycken EV, De Vos DE, Vanderleyden J, De Keersmaecker SC.** 2011. Structure-activity relationship of 4(5)-aryl-2-amino-1H-imidazoles, N1-substituted 2-aminoimidazoles and imidazo[1,2-a]pyrimidinium salts as inhibitors of biofilm formation by *Salmonella* Typhimurium and *Pseudomonas aeruginosa*. J Med Chem **54:**472-484.
2. **Steenackers HP, Ermolat'ev DS, Savaliya B, Weerdt AD, Coster DD, Shah A, Van der Eycken EV, De Vos DE, Vanderleyden J, De Keersmaecker SC.** 2011. Structure-activity relationship of 2-hydroxy-2-aryl-2,3-dihydro-imidazo[1,2-a]pyrimidinium salts and 2N-substituted 4(5)-aryl-2-amino-1H-imidazoles as inhibitors of biofilm formation by *Salmonella* Typhimurium and *Pseudomonas aeruginosa*. Bioorg Med Chem **19:**3462-3473.
3. **Steenackers H, Ermolat'ev D, Trang TT, Savalia B, Sharma UK, De Weerdt A, Shah A, Vanderleyden J, Van der Eycken EV.** 2014. Microwave-assisted one-pot synthesis and anti-biofilm activity of 2-amino-1H-imidazole/triazole conjugates. Org Biomol Chem **12:**3671-3678.
